# Efficacy of Binary Ethylenimine in the Inactivation of Foot-and-Mouth Disease Virus for Vaccine Production in South Korea

**DOI:** 10.3390/pathogens12060760

**Published:** 2023-05-25

**Authors:** Jae Young Kim, Sun Young Park, Jong Sook Jin, Dohyun Kim, Jong-Hyeon Park, Sang Hyun Park, Young-Joon Ko

**Affiliations:** Center for FMD Vaccine Research, Animal and Plant Quarantine Agency, 177 Hyeoksin-8-ro, Gimcheon-si 39660, Republic of Korea; ivorikim@korea.kr (J.Y.K.); sun3730@korea.kr (S.Y.P.); in75724@korea.kr (J.S.J.); doh936@korea.kr (D.K.); parkjhvet@korea.kr (J.-H.P.)

**Keywords:** FMDV, BEI, vaccine

## Abstract

Foot-and-mouth disease (FMD) vaccines must be produced in a biosafety level 3 facility, so the FMD virus (FMDV) must be completely inactivated after amplification. The inactivation kinetics of FMDV during vaccine antigen production were assessed by evaluating whether the viral titer dropped below 10^−7^ TCID_50_/mL within 24 h of binary ethyleneimine (BEI) treatment. This study dealt with four FMD vaccine candidate strains for the efficacy of BEI treatment at different concentrations and temperatures to determine the optimal inactivation condition of each virus. Two domestic isolates, O/SKR/Boeun/2017 (O BE) and A/SKR/Yeoncheon/2017 (A YC), and two recombinant viruses, PAK/44/2008 (O PA-2) and A22/Iraq/24/64 (A22 IRQ), were investigated. The O BE and A22 IRQ required 2 mM BEI at 26 °C and 0.5 mM BEI at 37 °C for complete inactivation. The O PA-2 and A YC required 2 mM BEI at 26 °C and 1 mM BEI at 37 °C. Crucially, the yield of FMD virus particles (146S) in the viral infection supernatant was higher (>4.0 µg/mL) than those previously reported; additionally, there was little antigen loss, even after 24 h of treatment with 3 mM BEI. Overall, it is considered economical to produce FMD vaccines using these four kinds of viruses; therefore, these candidate strains will be prioritized for the manufacture of FMD vaccines in South Korea.

## 1. Introduction

Foot-and-mouth disease (FMD) is a highly contagious vesicular disease that affects cloven-hoofed animals, such as cattle and pigs, and often causes extensive economic damage to the livestock industry [1].

FMD virus (FMDV), the corresponding causative agent of FMD, belongs to the *Aphthovirus* genus of the *Picornaviridae* family [2]. FMDV has a positive-sense, single-stranded RNA genome that is translated into a polyprotein, which is cleaved into structural proteins and non-structural proteins [3,4,5].

Vaccination is the best strategy to control FMD outbreaks, considering its highly contagious characteristics, such as airborne transmission. FMD vaccines must be produced in a biosafety level 3 facility, so the FMDV must be completely inactivated after amplification. Initially, formaldehyde was used for this inactivation. Then, in the 1950s, it was reported that formaldehyde did not inactivate FMDV during the first-order reaction [6]. An inactivating agent precursor, bromoethylamine hydrobromide (BEA)—an aziridine with significantly reduced toxicity compared to other aziridines—was developed in 1973; then, a Brazilian scientist introduced a method for the efficient production of the inactivating agent, binary ethyleneimine (BEI), using the BEA precursor [7]. Since the 1980s, most FMD vaccine manufacturers have used BEI as the inactivating agent [8].

FMD outbreaks have occurred several times in Korea since 2000, and the FMD vaccine has been administered to ungulates nationwide since December 2010. Because there is no domestic FMD vaccine, all FMD vaccines are imported. To localize the FMD vaccine in the near future, we have developed vaccine candidate strains using FMDVs obtained in South Korea and internationally. In the current study, we evaluated whether four representative vaccine candidate strains of FMDV could be effectively inactivated by BEI.

## 2. Materials and Methods

### 2.1. Cells and Viruses

BHK-21 suspension cells were established in serum-free media by the Animal and Plant Quarantine Agency (APQA) and the Korea Research Institute of Bioscience and Biotechnology; these cells were adapted for growth in Cellvento^TM^ BHK-200 cell culture medium (Merck, Darmstadt, Germany) with incubation at 110 rpm in a shaking incubator at 37 °C with 5% CO_2_. Cell number and viability were analyzed using the trypan blue exclusion method with an automated cell counter (Vi-Cell XR; Beckman Coulter Inc., Brea, CA, USA). A 70% volume of the total cells with 3 × 10^5^ cells/mL was grown for 3.5 days up to approximately 3 × 10^6^ cells/mL, and a 30% volume of fresh Cellvento medium was added without removing the spent media, followed by inoculation of FMDV into a flask.

Four FMD vaccine candidate strains were used to determine inactivation kinetics in this study. The two domestic isolates used were O/SKR/Boeun/2017 (O BE) [9] and A/SKR/Yeoncheon/2017 (A YC) [10]. The other two FMDVs were recombinant viruses with P1 replacement of the recombinant O1 Manisa backbone with P1 of the O/PAK/44/2008 and A22/Iraq/24/64, producing O PA-2 and A22 IRQ, respectively [11]. 

All four FMDV strains were inoculated into BHK-21 cell suspensions, and vaccine antigens were produced according to their optimized production conditions. The O BE and PA-2 strains were inoculated at a multiplicity of infection (MOI) of 0.001 and the viruses were harvested 16 h post-infection (hpi). A YC was inoculated at an MOI of 0.001 and harvested at 12 hpi. A22 IRQ was inoculated at an MOI of 0.001 and the virus was harvested at 24 hpi. Subsequently, the viral infection supernatants were collected using centrifugation (4000× *g*, 20 min, 4 °C). These supernatants were used to quantify FMDV particles (146S) and viral titers.

### 2.2. Preparation of BEI Solution

BEI was prepared by dissolving BEA in 10 mL of 0.2 N sodium hydroxide solution (Sigma-Aldrich, St. Louis, MO, USA) to a concentration of 0.1 M. The solution was then incubated at 37 °C in a shaking incubator at 100 rpm for 1 h. The pH of the solution was subsequently adjusted to a range of 8.5–9. This solution was prepared prior to each use. 

### 2.3. BEI Inactivation of FMDV Samples

For each sample, 100 mL of the FMDV supernatant was inactivated by adding various concentrations of BEI (0.5–3.0 mM). The supernatant was then incubated in a shaking incubator at 75 rpm at 26 °C and 37 °C for 24 h. Next, 12 mL samples were collected at hourly intervals up to 6 and 24 h after BEI treatment. Residual BEI was neutralized with a 10% volume of 1 M sodium thiosulfate (Daejung Chemicals, Siheung-si, Republic of Korea) to a final concentration of 2%. 

### 2.4. Quantification of FMDV Particles 

The harvested viral infection supernatant was treated with chloroform (Merck KGaA, Darmstadt, Germany) at a 1:1 (*v*/*v*) ratio and mixed via vigorous inversion for 5 min. The mixture was centrifuged at 3000× *g* for 15 min at 4 °C; then, the aqueous phase on top of the organic solvent was collected. After the chloroform treatment was conducted twice, the samples were centrifuged at 16,000× *g* for 10 min, and the supernatant was collected. The samples were treated with benzonase (Sigma-Aldrich) at a final concentration of 0.025 units/μL, followed by shaking incubation at 37 °C for 1 h. After digestion, the samples were centrifuged at 16,000× *g* for 10 min at 4 °C to obtain a clear supernatant. Subsequently, samples were filtered through 0.22 μm Millex-GV filters (Merck KGaA, Darmstadt, Germany) and loaded into size-exclusion high-performance liquid chromatography (SE-HPLC) columns to quantify the 146S intact viral particle in samples. 146S particles were quantified using SE-HPLC. This analysis was performed on a TSKgel G4000PWXL (300 mm × 7.8 mm) column (TOSOH Bioscience, Tokyo, Japan) combined with a TSKgel PWXL Guardcol (40 mm × 6.0 mm) guard column (TOSOH Bioscience) using an Agilent 1260 Infinity II system (Agilent Technologies, Santa Clara, CA, USA). The mobile phase was composed of 30 mM Tris-HCl and 400 mM NaCl (pH 8.0) and the flow rate was set at 0.5 mL/min. The area under the target peak was integrated using the OpenLAB chromatography data system (CDS) ChemStation Edition Rev. C01.10 and the quantity of 146S antigens (µg/mL) was calculated according to a previous study [12].

### 2.5. Virus Titration

Viral titers were determined using adherent BHK-21 cells via endpoint titration using the Spearman–Kärber calculation; these were presented as the tissue culture infectious dose that affected 50% of the culture (TCID_50_) per milliliter. 

### 2.6. Data Aanalysis 

Each experiment was conducted three times, and the mean and standard deviation of all values were described. BEI inactivation kinetics, including viral titers and inactivation time intervals, were analyzed using GraphPad Prism version 9 (GraphPad Software, La Jolla, CA, USA) for visual representations.

## 3. Results

### 3.1. BEI Inactivation Kinetics

**Figure 1:**
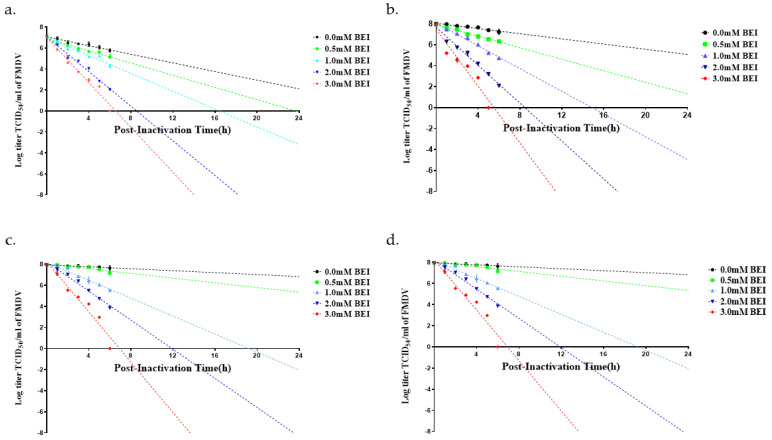
Inactivation kinetics of four FMD vaccine candidate strains at 26 °C. The viral infection supernatant was inactivated by binary ethyleneimine (BEI) treatment, with samples taken hourly up to 6 and 24 h. The strains assessed included: (**a**) O/SKR/Boeun/2017 (O BE), (**b**) PAK/44/2008 (O PA−2), (**c**) A/SKR/Yeoncheon/2017 (A YC), and (**d**) A22/Iraq/24/64 (A22 IRQ).

**Figure 2:**
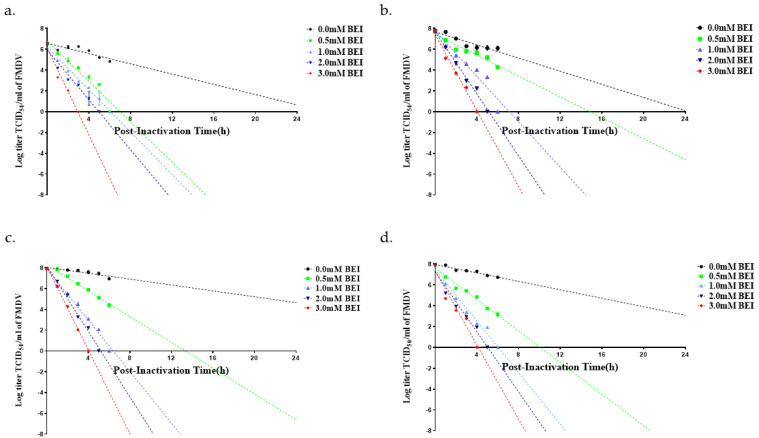
Inactivation kinetics of four FMD vaccine candidate strains at 37 °C. The viral infection supernatant was inactivated by BEI treatment, with samples taken hourly up to 6 and 24 h. The strains assessed included: (**a**) O BE, (**b**) O PA−2, (**c**) A YC, and (**d**) A22 IRQ.

During virus inactivation, samples were collected hourly from 0 to 6 h and then 24 h post-inactivation to monitor the rate and linearity of the inactivation process. The viral titers before inactivation were approximately 7 log TCID_50_/mL for O BE and approximately 8 log TCID_50_/mL for the other three FMD viruses (Figure 1 and Figure 2). Viral titers were measured for virus samples inactivated at 26 °C and 37 °C with different concentrations (0.5–3.0 mM) of BEI. The corresponding results demonstrated that the viral titer decreased faster at 37 °C than at 26 °C when treated with the same concentration of BEI. In addition, the viral titer decreased rapidly as the BEI concentration increased, regardless of temperature.

### 3.2. Effects of Temperature and BEI Concentration

The criterion for inactivation of FMDV is a decrease in the viral titer to −7 log TCID_50_/mL within 24 h of BEI treatment. When the virus inactivation was performed at 26 °C, all four viruses treated with 2 mM BEI exhibited a decrease in the corresponding viral titer to −7 log TCID_50_/mL within 24 h. However, treatment with 0.5 and 1.0 mM BEI was not consistently effective in the inactivation of FMDV and did not reduce virus titers to −7 log TCID_50_/mL within 24 h and 26 °C (Table 1 and Figure 1). When the inactivation of FMDV was performed at 37 °C, O BE and A22 IRQ exhibited a decrease in viral titer to −7 log TCID_50_/mL within 24 h post-inactivation with 0.5 mM BEI (Table 1 and Figure 2). In contrast, O PA-2 and A YC exhibited a decrease in viral titers to −7 log TCID_50_/mL within 24 h post-inactivation with 1.0 mM BEI when incubated at 37 °C. 

### 3.3. Effects of BEI Inactivation on FMDV Particle (146S) Yield

Because the FMD vaccine antigen can be lost during the inactivation phase of FMDV, we also investigated changes in the vaccine antigen yield depending on the inactivation conditions (Table 2). The 146S content of FMDV was measured by SE-HPLC at 0, 6, and 24 h after BEI treatment at 26 °C and 37 °C. For the O BE strain, there was no loss from the initial antigen yield of 9.58 µg/mL, regardless of the inactivation temperature and BEI concentration. O PA-2 exhibited a 97.8% antigen recovery after 24 h with 2 mM BEI and a 96.3% antigen recovery after 24 h with 3 mM BEI when this virus was inactivated at 26 °C with an initial antigen yield of 6.92 µg/mL. On the other hand, when the O PA-2 was inactivated at 37 °C, the antigen recovery rates were 97.8% and 95.9% 24 h after treatment with 1 mM and 3 mM BEI, respectively. When A YC, with an initial antigen yield of 8.0 µg/mL, was inactivated at 26 °C, the antigen recovery rates were 98.3%, 96.7%, 97.1%, and 98.8% 24 h after treatment with 0.5, 1, 2, and 3 mM BEI, respectively. On the other hand, inactivation of this strain at 37 °C resulted in an antigen recovery of 98.8% and 97.5% 24 h after treatment with 2 mM and 3 mM BEI, respectively. Finally, for A22 IRQ, there was no loss from the initial antigen yield of 4.3 µg/mL regardless of the inactivation temperature and BEI concentration.

## 4. Discussion

A FMD vaccine should meet these three requirements. First, the vaccine must be effective in protecting against the FMDV. Second, the non-structural FMDV proteins must be removed from the production process to differentiate between infected and vaccinated animals using a serological assay. Third, the inactivation of FMDV by BEI must be validated and documented to show the corresponding inactivation kinetics [13]. Therefore, this study aimed to evaluate the BEI-dependent inactivation of four representative vaccine candidate strains that could be used to manufacture FMD vaccines in South Korea in the near future. 

In this study, we inactivated four kinds of FMD vaccine candidate strains at two temperatures, 37 °C and 26 °C, for several reasons. Typically, this virus is propagated and inactivated at 37 °C. Nonetheless, there is a rationale behind comparing the viral inactivation of FMDV at 26 °C and 37 °C; specifically, FMD vaccine antigen recovery may be reduced by enzyme-dependent degradation in the cell when exposed to 37 °C for a long period [14]. In addition, the 2009 edition of the OIE manual states that inactivation of FMDV could be performed at 26 °C and 37 °C. Based on this, we compared the inactivation of four FMDVs at these two temperatures across various BEI concentrations to allow the selection of optimal inactivation conditions that did not affect vaccine antigen recovery during BEI treatment.

The in-process quality control of the inactivation kinetics of FMDV was conducted as follows: the log_10_ infectivity of the timed samples was plotted against time, and the inactivation procedure was not considered satisfactory unless at least the latter part of the slope of the line was linear; additionally, extrapolation was used to ensure that there would be < 1 infectious particle per 10^4^ L of liquid preparation at the end of this inactivation period [13].

Based on these guidelines, inactivation kinetics were determined for the four FMD vaccine candidate strains. The O BE and A22 IRQ strains required 2 mM BEI at 26 °C and 0.5 mM BEI at 37 °C to be completely inactivated. Alternatively, the O PA-2 and A YC strains required 2 mM BEI at 26 °C and 1.0 mM BEI at 37 °C for completely inactivation. 

The results observed in this study in which the viral titer decreased rapidly with increasing BEI concentration and in which the viruses were inactivated earlier at 37 °C than at 26 °C aligned with those in previous reports [15,16,17,18].

What distinguishes this study from other, similar reports is that the yield of FMDV particles (146S) obtained from these viral infection supernatants was higher than those previously reported; further, there was almost no loss of antigen recovery observed in the current study after 24 h of inactivation with 3 mM BEI concentration at 37 °C. In this study, the O BE, O PA-2, A YC, and A22 IRQ viral infection supernatants contained 9.58, 6.92, 8.0, and 4.30 µg/mL of antigen, respectively. In contrast, previous reports have shown initial antigen levels of approximately 1 µg/mL in viral infection supernatants prior to inactivation with BEI treatment [15,16,19]. In studies conducted for different purposes, Li et al. reported an approximate 3 µg/mL antigen recovery in the corresponding viral infection supernatant [20] and Barteling and Meroen reported a 2.6 µg/mL antigen recovery [21].

In contrast to the high stability of our four vaccine candidate strains in this study, a prior study reported that >60% of the antigen was lost after 24 h of treatment with 1 mM BEI at 37 °C [17]. In addition, the O1 Campos viral strain exhibited an antigen loss > 80% following incubation at 37 °C for 24 h without BEI treatment [22].

In conclusion, the four FMD vaccine candidate strains in the current study retained a high antigen yield (approximately 4–10 µg/mL). These viruses were easily inactivated with low concentrations of BEI and did not exhibit antigen loss, even after inactivation with 3 mM BEI for 24 h. Consequently, this indicates that these FMDV strains are highly economical candidates for use in commercial vaccines. Overall, South Korea plans to manufacture an FMD vaccine in the near future and these candidate vaccine strains are likely to be the first choice in this process. 

## Figures and Tables

**Table 1:** Time taken to reach <10^−7^ TCID_50_/mL after BEI treatment, determined using regression curve analysis.

BEI	0.5 mM		1.0 mM		2.0 mM		3.0 mM	
Temperature	26 °C	37 °C	26 °C	37 °C	26 °C	37 °C	26 °C	37 °C
O BE	48 h	14 h	32 h	12 h	17 h	11 h	13 h	6 h
O PA-2	54 h	28 h	28 h	14 h	16 h	10 h	8 h	13 h
A YC	134 h	25 h	36 h	12 h	22 h	10 h	12 h	8 h
A22 IRQ	68 h	19 h	30 h	12 h	20 h	10 h	13 h	8 h

**Table 2:** The amount of FMDV 146S antigen in viral infection supernatants after BEI treatment at 26 °C and 37 °C for 6 and 24 h.

Virus		26 °C					37 °C				
	Time (h)	0 mM BEI	0.5 mM BEI	1 mM BEI	2 mM BEI	3 mM BEI	0 mM BEI	0.5 mM BEI	1 mM BEI	2 mM BEI	3 mM BEI
O BE	0	9.58 ± 0.23 (100)	9.58 ± 0.23 (100)	9.58 ± 0.23 (100)	9.58 ± 0.23 (100)	9.58 ± 0.23 (100)	9.58 ± 0.23 (100)	9.58 ± 0.23 (100)	9.58 ± 0.23 (100)	9.58 ± 0.23 (100)	9.58 ± 0.23 (100)
	6	9.83 ± 0.40 (102.6)	9.87 ± 0.25 (102.9)	9.87 ± 0.21 (102.9)	9.80 ± 0.22 (102.3)	9.50 ± 0.28 (99.2)	10.07 ± 0.09 (105.1)	10.37 ± 0.45 (108.2)	10.03 ± 0.05 (104.7)	9.97 ± 0.05 (104.0)	10.07 ± 0.31 (105.1)
	24	10.17 ± 0.09 (105.8)	10.13 ± 0.19 (105.8)	9.83 ± 0.26 (102.6)	9.83 ± 0.12 (102.6)	9.63 ± 0.09 (100.6)	10.10 (105.4)	10.03 ± 0.12 (104.7)	10.07 ± 0.05 (105.1)	9.90 ± 0.08 (103.3)	9.77 ± 0.05 (102.0)
O PA-2	0	6.92 ± 0.17 (100)	6.92 ± 0.17 (100)	6.92 ± 0.17 (100)	6.92 ± 0.17 (100)	6.92 ± 0.17 (100)	6.92 ± 0.17 (100)	6.92 ± 0.17 (100)	6.92 ± 0.17 (100)	6.92 ± 0.17 (100)	6.92 ± 0.17 (100)
	6	7.03 ± 0.05 (101.6)	7.33 ± 0.05 (106.0)	7.27 ± 0.05 (105.0)	7.10 (102.6)	6.70 ± 0.08 (96.8)	7.07 ± 0.05 (102.1)	7.03 ± 0.12 (101.6)	7.20 ± 0.08 (104.0)	7.23 ± 0.05 (104.5)	6.77 ± 0.05 (97.8)
	24	6.97 ± 0.05 (100.7)	7.00 ± 0.08 (101.2)	6.97 ± 0.09 (100.7)	6.77 ± 0.05 (97.8)	6.67 ± 0.05 (96.3)	6.90 ± 0.29 (99.7)	7.10 (102.6)	6.77 ± 0.09 (97.8)	7.03 ± 0.12 (101.6)	6.63 ± 0.05 (95.9)
A YC	0	8.00 ± 0.12 (100)	8.00 ± 0.12 (100)	8.00 ± 0.12 (100)	8.00 ± 0.12 (100)	8.00 ± 0.12 (100)	8.00 ± 0.12 (100)	8.00 ± 0.12 (100)	8.00 ± 0.12 (100)	8.00 ± 0.12 (100)	8.00 ± 0.12 (100)
	6	8.27 ± 0.05 (103.3)	8.27 ± 0.05 (103.3)	8.30 ± 0.08 (103.8)	8.07 ± 0.09 (100.8)	8.10 ± 0.08 (101.3)	8.50 ± 0.16 (106.3)	8.47 ± 0.05 (105.8)	8.53 ± 0.21 (106.7)	8.27 ± 0.09 (103.3)	8.13 ± 0.12 (101.7)
	24	8.30 (103.8)	7.87 ± 0.12 (98.3)	7.73 ± 0.05 (96.7)	7.77 ± 0.05 (97.1)	7.90 ± 0.08 (98.8)	8.33 ± 0.05 (104.2)	8.20 ± 0.08 (102.5)	8.00 ± 0.16 (100)	7.90 ± 0.16 (98.8)	7.80 (97.5)
A22 IRQ	0	4.30 ± 0.06 (100)	4.30 ± 0.06 (100)	4.30 ± 0.06 (100)	4.30 ± 0.06 (100)	4.30 ± 0.06 (100)	4.30 ± 0.06 (100)	4.30 ± 0.06 (100)	4.30 ± 0.06 (100)	4.30 ± 0.06 (100)	4.30 ± 0.06 (100)
	6	4.77 ± 0.19 (110.9)	4.67 ± 0.12 (108.5)	4.63 ± 0.21 (107.8)	4.47 ± 0.12 (103.9)	4.37 ± 0.21 (101.6)	4.47 ± 0.12 (103.9)	4.40 (102.3)	4.30 ± 0.14 (100)	4.43 ± 0.05 (103.1)	4.23 ± 0.12 (98.4)
	24	4.57 ± 0.09 (106.2)	4.43 ± 0.05 (103.1)	4.37 ± 0.17 (101.6)	4.37 ± 0.05 (101.6)	4.33 ± 0.09 (100.8)	4.47 ± 0.12 (103.9)	4.67 ± 0.05 (108.5)	4.57 ± 0.09 (106.2)	4.47 ± 0.09 (103.9)	4.53 ± 0.05 (105.4)

FMDV 146S antigen amount is represented as the mean ± standard deviation (SD). The number in parentheses is the percentage value calculated based on the amount of antigen before BEI treatment. The experiment was performed in triplicate.

## Data Availability

Not applicable.

## References

[1] Knight-Jones T.J., Rushton J. (2013). The economic impacts of foot and mouth disease—What are they, how big are they and where do they occur?. Prev. Vet. Med..

[2] Sharma G.K., Mohapatra J.K., Mahajan S., Matura R., Subramaniam S., Pattnaik B. (2014). Comparative evaluation of non-structural protein-antibody detecting ELISAs for foot-and-mouth disease sero-surveillance under intensive vaccination. J. Virol. Methods.

[3] Grubman M.J., Baxt B. (2004). Foot-and-mouth disease. Clin. Microbiol. Rev..

[4] Roland R., Rueckert E.W. (1984). Systematic Nomenclature of Picornavirus Proteins. J. Virol..

[5] Liu Z., Shao J., Zhao F., Zhou G., Gao S., Liu W., Lv J., Li X., Li Y., Chang H. (2017). Chemiluminescence Immunoassay for the Detection of Antibodies against the 2C and 3ABC Nonstructural Proteins Induced by Infecting Pigs with Foot-and-Mouth Disease Virus. Clin. Vaccine Immunol..

[6] Lombard M., Pastoret P.P., Moulin A.M. (2007). A brief history of vaccines and vaccination. Rev. Sci. Tech..

[7] Barteling S.J. (2002). Development and performance of inactivated vaccines against foot and mouth disease. Rev. Sci. Tech..

[8] Bahnemann H.G. (1975). Binary ethylenimine as an inactivant for foot-and-mouth disease virus and its application for vaccine production. Arch. Virol..

[9] Lee G., Hwang J.H., Park J.H., Lee M.J., Kim B., Kim S.M. (2020). Vaccine strain of O/ME-SA/Ind-2001e of foot-and-mouth disease virus provides high immunogenicity and broad antigenic coverage. Antivir. Res..

[10] Hwang J.H., Lee G., Kim A., Park J.H., Lee M.J., Kim B., Kim S.M. (2021). A Vaccine Strain of the A/ASIA/Sea-97 Lineage of Foot-and-Mouth Disease Virus with a Single Amino Acid Substitution in the P1 Region That Is Adapted to Suspension Culture Provides High Immunogenicity. Vaccines.

[11] Lee S.-Y., Lee Y.-J., Kim R.-H., Park J.-N., Park M.-E., Ko M.-K., Choi J.-H., Chu J.-Q., Lee K.-N., Kim S.-M. (2017). Rapid Engineering of Foot-and-Mouth Disease Vaccine and Challenge Viruses. J. Virol..

[12] Spitteler M.A., Romo A., Magi N., Seo M.G., Yun S.J., Barroumeres F., Regulier E.G., Bellinzoni R. (2019). Validation of a high performance liquid chromatography method for quantitation of foot-and-mouth disease virus antigen in vaccines and vaccine manufacturing. Vaccine.

[13] WOAH (2022). Chapter 3. 1. 8. Foot and Mouth Disease (Infection with Foot and Mouth Disease Virus). Manual of Diagnostic Tests and Vaccines for Terrestrial Animals. https://www.woah.org/en/what-we-do/standards/codes-and-manuals/terrestrial-manual-online-access/.

[14] Bahnemann H.G. (1990). Inactivation of viral antigens for vaccine preparation with particular reference to the application of binary ethylenimine. Vaccine.

[15] Wu P., Rodriguez Y.Y., Hershey B.J., Tadassa Y., Dodd K.A., Jia W. (2021). Validation of a binary ethylenimine (BEI) inactivation procedure for biosafety treatment of foot-and-mouth disease viruses (FMDV), vesicular stomatitis viruses (VSV), and swine vesicular disease virus (SVDV). Vet. Microbiol..

[16] Aarthi D., Ananda Rao K., Robinson R., Srinivasan V.A. (2004). Validation of binary ethyleneimine (BEI) used as an inactivant for foot and mouth disease tissue culture vaccine. Biologicals.

[17] Sarkar A., Tamil Selvan R.P., Kishore S., Ganesh K., Bhanuprakash V. (2017). Comparison of different inactivation methods on the stability of Indian vaccine strains of foot and mouth disease virus. Biologicals.

[18] Ismail A.H., El-Mahdy S.A., Mossad W.G., Abd El-Krim A.S., Abou El-Yazid M., Ali S.M. (2013). Optimization of the Inactivation Process of FMD Virus Serotype SAT-2 by Binary Ethyleneimine (BEI). J. Vet. Adv..

[19] Rweyemamu M.M., Unehara O., Giorgi W., Medeiros R., Lucca D., Baltazar M. (1989). Effect of formaldehyde and binary ethyleneimine (BEI) on the integrity of foot and mouth disease virus capsid. Rev. Sci. Tech..

[20] Li X.R., Yang Y.K., Wang R.B., An F.L., Zhang Y.D., Nie J.Q., Ahamada H., Liu X.X., Liu C.L., Deng Y. (2019). A scale-down model of 4000-L cell culture process for inactivated foot-and-mouth disease vaccine production. Vaccine.

[21] Barteling S.J., Meloen R.H. (1974). A simple method for the quantification of 140 s particles of foot-and-mouth disease virus (FMDV). Arch. Für Die Gesamte Virusforsch..

[22] Bucafusco D., Di Giacomo S., Pega J., Schammas J.M., Cardoso N., Capozzo A.V., Perez-Filgueira M. (2015). Foot-and-mouth disease vaccination induces cross-reactive IFN-γ responses in cattle that are dependent on the integrity of the 140S particles. Virology.

